# Understanding mechanisms of vitiligo development in Smyth line of chickens by transcriptomic microarray analysis of evolving autoimmune lesions

**DOI:** 10.1186/1471-2172-13-18

**Published:** 2012-04-13

**Authors:** Fengying Shi, Byung-Whi Kong, Joon Jin Song, Jeong Yoon Lee, Robert L Dienglewicz, Gisela F Erf

**Affiliations:** 1 Center of Excellence for Poultry Science, University of Arkansas, Division of Agriculture, Fayetteville, AR 72701, USA; 2 Department of Mathematical Sciences, Center for Statistical Research and Consulting, University of Arkansas, Fayetteville, AR 72701, USA

## Abstract

**Background:**

The Smyth line (SL) of chicken is an excellent avian model for human autoimmune vitiligo. The etiology of vitiligo is complicated and far from clear. In order to better understand critical components leading to vitiligo development, cDNA microarray technology was used to compare gene expression profiles in the target tissue (the growing feather) of SL chickens at different vitiligo (SLV) states.

**Results:**

Compared to the reference sample, which was from Brown line chickens (the parental control), 395, 522, 524 and 526 out of the 44 k genes were differentially expressed (DE) (P ≤ 0.05) in feather samples collected from SL chickens that never developed SLV (NV), from SLV chickens prior to SLV onset (EV), during active loss of pigmentation (AV), and after complete loss of melanocytes (CV). Comparisons of gene expression levels within SL samples (NV, EV, AV and CV) revealed 206 DE genes, which could be categorized into immune system-, melanocyte-, stress-, and apoptosis-related genes based on the biological functions of their corresponding proteins. The autoimmune nature of SLV was supported by predominant presence of immune system related DE genes and their remarkably elevated expression in AV samples compared to NV, EV and/or CV samples. Melanocyte loss was confirmed by decreased expression of genes for melanocyte related proteins in AV and CV samples compared to NV and EV samples. In addition, SLV development was also accompanied by altered expression of genes associated with disturbed redox status and apoptosis. Ingenuity Pathway Analysis of DE genes provided functional interpretations involving but not limited to innate and adaptive immune response, oxidative stress and cell death.

**Conclusions:**

The microarray results provided comprehensive information at the transcriptome level supporting the multifactorial etiology of vitiligo, where together with apparent inflammatory/innate immune activity and oxidative stress, the adaptive immune response plays a predominant role in melanocyte loss.

## Background

Vitiligo is a postnatal hypopigmentary disease characterized by appearance of white patches due to loss of functioning pigment producing cells (melanocytes) in affected skin. Vitiligo renders patients more susceptible to various forms of skin cancer and has negative psycho-social effects on life quality of vitiligo patients, especially when the depigmented skin is present in sun-exposed areas [1,2]. The etiology of vitiligo is multifaceted, involving genetic, immune, metabolic and environmental factors and the loss of melanocytes in affected tissues is generally considered to result from autoimmune destruction of melanocytes [3].

The Smyth line (SL) of chicken is an excellent animal model for human autoimmune vitiligo, for which many phenotypical and etiological similarities to human vitiligo have been documented [4-6]. The genetically vitiligo-susceptible SL chickens [7-9] develop postnatal and spontaneous autoimmune loss of feather pigmentation (SLV), with 80-95% of hatch-mates expressing the disorder during early adulthood [5,8]. Etiopathologically, SLV development and progression is associated with mononuclear leukocyte infiltration into the target tissue [6], melanocyte-specific humoral [10-12] and cell-mediated immunity [13-17] and elevated levels of oxidative stress [18]. In addition, routine vaccination at hatch with live herpesvirus of turkey against lymphoma-causing Marek's disease virus was identified as a dependable environmental factor related to SLV expression [19]. Taken together, like in humans, SLV is a multifactorial disease, which underlines the suitability of the SL chicken as the avian model for human vitiligo.

Several vitiligo-susceptibility genes have been identified for human vitiligo using the candidate gene approach, the genome-wide linkage approach and the gene expression approach [20]. However, identification of susceptibility genes in humans was complicated by the tremendous difference in genetic background among humans [21]. By contrast, SL chickens are highly inbred [22] and share the same MHC-haplotype and background genes as the Brown line (BL) of chicken from which the SL was derived. Together the vitiligo-expressing SL chicken and its parental BL control provide an excellent model to focus research efforts on vitiligo-related aspects and activities. Additional attributes of the SL chicken model that make it highly suitable for vitiligo study are the predictably high incidence of vitiligo expression (80-95%) [5] and the fact that melanocytes are located in growing feathers [8]. Collection of growing feathers is a minimally invasive procedure and growing feathers regenerate, providing opportunity to repeatedly sample and study the spontaneously evolving lesions in the same individual throughout the natural process of vitiligo development.

Using the SL chicken vitiligo model, the purpose of this study was to examine gene-expression associated with vitiligo development by comparing transcriptomic profiles in samples collected from SL chickens that never developed SLV (NV) and those collected from the same SLV chickens before vitiligo onset (EV), during active (AV) and after complete loss of pigmentation (CV). The results from the current study will further shed light on the critical events related to vitiligo development in SL chickens and in humans.

## Methods

### Animals

Eighteen SL and four MHC (B^*101/101*^)- and age-matched BL chicks were randomly selected from 15 SL and 4 BL families. The BL chicken is the parental control from which the SL chicken was derived. This line continues to have a rare incidence of vitiligo (< 2%). One-day old chicks were vaccinated with live herpesvirus of turkey (Fort Dodge Animal Health, Fort Dodge, Iowa) to protect against Marek's disease. All chicks were kept in one floor pen at the Arkansas Experiment Station Poultry Farm in Fayetteville, AR, USA with feed and water provided ad libitum. The animal use was approved by the University of Arkansas Institutional Animal Care and Use Committee.

### Feather collection

The living part of growing feathers (feather tip) [6] was collected twice a week starting from 4 weeks of age until the chickens were 20 weeks old (young adulthood; natural endpoint when feather growth is complete). For each chicken and collection, three feather tips were snap frozen with liquid nitrogen in Tissue-Tek^® ^O.C.T freezing medium (Sakura Finetek Inc., Torrance, CA). All samples were kept at -80°C until use. For each chicken, vitiligo development was evaluated at each feather collection based on pigmentation loss in the growing feathers using a scale of 1 to 5 following previously described criteria [16].

### Feather sample selection for the microarray study

By the end of the 20-week feather collection, 12 of the SL chickens had developed SLV (SLV chickens) while the other 6 SL (non-vitiliginous SL chickens) and the 4 BL chickens had not developed vitiligo. To represent various stages of SLV development (early, active, and complete vitiligo) feather tips from each of the 12 SLV chickens were selected as follows for RNA isolation and down-stream analysis: two collections before (within 1 week) visible vitiligo onset (early vitiligo; EV sample; vitiligo score 1), two collections during active vitiligo (AV sample; vitiligo score 2-4; partially depigmented regenerating feathers were selected), and two collections at least one week after complete depigmentation (CV sample; vitiligo score 5). For the 6 non-vitiliginous SL (NV sample, vitiligo score 1) and the 4 non-vitiliginous BL control chickens (BL sample), feather tips obtained at the same age when the selected SLV samples were collected (age-matched) were chosen for RNA isolation.

### RNA isolation and quantification

Feather tips were taken out of O. C. T. blocks and homogenized by Tissue Tearor™ (Biospec Products, Inc. Model: 985370-395) at -20°C in lysis buffer provided by Qiagen RNeasy^® ^Mini kit (Qiagen Inc., Valencia, CA). Total RNA was isolated from homogenates following manufacturer's instruction with on-column DNA digestion (Qiagen Inc., Valencia, CA). RNA was eluted in 30 μl RNase-, DNase-free water and stored at -80°C until use. RNA integrity and concentration were determined with an Experion™ automated electrophoresis system and Experion™ RNA StdSens analysis kit (Bio-Rad laboratories, Hercules, CA) following manufacturer's instruction.

### Making RNA pools for microarray analysis

For microarray analysis, separate pools of RNA were prepared for NV, EV, AV, CV and BL samples by mixing equal amounts of RNA from the pre-selected feather tips of each bird in the respective samples. For this, the 12 vitiliginous and 6 non-vitiliginous SL chickens were evenly divided into 3 groups of 4 and 2 birds each, representing three SLV- and three NV- replications, respectively. For each SLV group, RNA pools of their individual EV, AV and CV samples were prepared. Using RNA isolated from the age-matched feather samples, one RNA pool was made for each NV group and one RNA pool was made for the BL control group. The final concentrations of RNA pools were determined by the Thermo Scientific NanoDrop™ 1000 Spectrophotometer (Thermo Scientific, Wilmington, DE, USA).

### Two-color microarray

A two-color labeling microarray system together with chicken 4 × 44 k Agilent gene expression microarray slides (array IDs: 251506810338, 251506810339 and 251506810340; Agilent Technologies, Inc., Santa Clara, CA) was employed to compare global transcriptomic profiles between NV, EV, AV and CV samples by using the BL sample as the reference (reference design). RNA pools from SL chickens (NV, EV, AV and CV) were labeled with Cy5 (fluoresces red) and the BL pool was labeled with Cy3 (fluoresces green) by incorporating cyanine 5- and cyanine 3-CTP, respectively, during cRNA generation using T7 RNA polymerase. The two-color microarray workflow from sample amplification and labeling to hybridization was carried out as previously described [23]

### Microarray data collection and analysis

Raw data were collected and analyzed with a GenePix 4000B scanner and GenePix Pro 6.1 Software (Molecular Devices, Inc., Sunnyvale, CA), respectively. Background-corrected red and green intensities for each spot were used in the subsequent analysis. Locally weighted linear regression (LOWESS) was employed to normalize the fluorescent intensities to retain only biological differences by removing undesirable systemic variations in microarray experiments. Moderated t-statistic based on the empirical Bayes method [24] was applied to each gene to identify differentially expressed genes in NV, EV, AV and CV samples relative to BL controls. Comparisons between NV, EV, AV and CV samples (within SL comparisons) were performed by One-way ANOVA test using JMP Genomics 4 (multiple means comparison) (SAS Institute Inc, Cary, NC). Genes with P ≤ 0.05 were considered differentially expressed (DE). Log2 data obtained by gene-expression analyses were transformed and reported as linear fold change values in the result section.

### Microarray validation with qRT-PCR

Eleven genes were selected for validation by Taqman^® ^qRT-PCR using the same RNA samples as in the microarray. Primers and probes were designed by Primer Express 3.0 (Applied Biosystems, Foster City, CA) and all primer and probe sets are available upon request. RNA (2 μg) from NV, EV, AV, CV, and BL pools was reverse-transcribed into cDNA in a 30 μl reaction volume using a High Capacity cDNA reverse transcription kit without RNase inhibitor according to the manufacturer's protocol (Applied Biosystems, Foster City, CA). The resulting cDNA was diluted with RNase- and DNase-free water at a ratio of 1:3. One μl of this product was subjected to PCR using Taqman 2X Universal PCR master mix with glyceraldehyde-3-phosphate dehydrogenase (GAPDH) as the endogenous control gene and the cDNA from the BL RNA pool as the calibrator. The following PCR conditions were used: 1 cycle at 50°C for 2 minutes, 95°C for 10 minutes, and 45 cycles of denaturation at 95°C for 15 seconds, and annealing/extension at 60°C for 60 seconds. The relative gene expression was determined by the delta delta Ct (ΔΔCt) method [25].

### Bioinformatics

DE genes were analyzed through the use of Ingenuity Pathways Analysis (IPA, Version 9.0; Ingenuity Systems^®^; http://www.ingenuity.com).

## Results

### Differentially expressed (DE, P ≤ 0.05) genes in SL samples (NV, EV, AV and CV) relative to BL control samples

Relative to the BL sample, 395, 522, 524 and 526 DE genes were identified for NV, EV, AV and CV samples, respectively, which gave rise to 151, 213, 212 and 212 DE genes for Ingenuity Pathways Analysis (IPA) after deletion of genes without information, genes from cDNA libraries and clones and genes related to hypothetical proteins. The top 10% of up- and down-regulated DE genes for individual SLV states are summarized in Table 1.

**Table 1:** Top 10% DE genes^a ^in growing feathers from SL chickens relative to non-vitiliginous parental BL controls

GeneName/Symbol	Up-regulated DE genes	GeneName/Symbol	Down-regulated DE genes
**NV^b^**			

IGJ	+5.99^c^	Lipoprotein VSAF	-11.51

CHIA	+5.29	MMP9	-7.81

similar to TCR delta chain	+3.67	similar to GHITM protein	-5.02

COBT	+3.58	TMEM22	-3.09

RSFR	+2.82	HVCN1	-3.00

CCL19	+2.79	HNRPU protein	-2.64

similar to CXCL13	+2.72	MMP13	-2.64

GBP	+2.69	similar to keratin	-2.49

MX	+2.64	FMO6	-2.46

similar to MMR (CD206)	+2.63	HIST2H3C protein	-2.45

similar to MSTP055	+2.61	ASB12	-2.38

similar to T-cell receptor gamma Vg2-Jg2	+2.60	altronate hydrolase	-2.35

GZMA	+2.60	GOLGB1	-2.34

TMED8	+2.53	RuBisCu	-2.27

ISG12-2	+2.50	LEPREL1	-2.21

**EV**			

similar to CXCL13	+16.01	Lipoprotein VSAF	-12.05

GBP	+12.08	MMP9	-5.15

LITAF	+10.08	similar to keratin	-5.11

TNFSF13B	+9.77	PTH	-4.88

similar to sodium channel alpha subunit	+8.74	Adenine phosphoribosyltransferase	-3.51

CCL19	+8.17	similar to GHITM protein	-3.44

GZMA	+7.41	HIST2H3C protein	-3.15

IL21R	+7.41	FMO6	-2.94

C3AR1	+7.15	TAC1	-2.83

CHIA	+7.03	KCNE1L	-2.82

similar to immune-responsive protein 1	+6.71	SLC22A5	-2.79

ISG12-2	+6.67	GOLGB1	-2.73

Chemokine ah294	+6.43	TMEM22	-2.70

MX	+6.17	MMP13	-2.64

TLR15	+6.15	FCGBP	-2.46

similar to MD-2	+6.12	HNRPU protein	-2.43

IGJ	+6.04	Cytochrome b	-2.38

Mucolipin 2	+6.03	similar to CLIC3	-2.34

Gp91-phox	+5.91	HVCN1	-2.34

similar to IFIT-5	+5.89	Probable G protein-coupled receptor 26	-2.27

IRF1	+5.85	Thrombomucin	-2.24

**AV**			

similar to sodium channel alpha subunit	+17.96	PTH	-11.70

IGJ	+17.84	Lipoprotein VSAF	-11.50

similar to CXCL13	+17.13	SLC24A5	-7.23

POU2AF1	+16.37	MMP9	-6.02

TNFSF13B	+16.01	MMP115	-5.57

LITAF	+14.92	similar to GHITM protein	-4.57

LECT2	+11.20	Adenine phosphoribosyltransferase	-4.38

similar to immune-responsive protein 1	+10.94	similar to keratin	-4.13

IL21R	+10.80	CRYAB	-3.78

Gp91-phox	+10.58	TMEM22	-3.61

Mucolipin 2	+10.58	KCNE1L	-3.46

C3AR1	+10.32	Shikimate 5-dehydrogenase	-3.39

GBP	+10.23	TAC1	-3.16

Myeloid antimicrobial peptide 27	+9.38	FMO6	-2.74

LYZ	+8.92	UPK3B	-2.62

similar to MD-2	+8.73	MRRF	-2.60

similar to regulator of G-protein signaling 1	+8.65	MMP13	-2.60

TLR15	+8.32	similar to ALS2	-2.49

CCL19	+7.72	HIST2H3C protein	-2.45

CHIA	+7.65	TYR	-2.42

C-C chemokine receptor 11 like	+7.59	SLC22A5	-2.42

**CV**			

IGJ	+20.76	SLC24A5	-17.06

POU2AF1	+18.19	TYR	-15.54

Myeloid antimicrobial peptide 27	+13.31	MMP115	-14.01

similar to sodium channel alpha subunit	+12.53	Lipoprotein VSAF	-12.43

LECT2	+11.91	PTH	-11.89

similar to CXCL13	+8.94	MMP9	-6.95

CHIA	+7.39	CRYAB	-5.09

TNFSF13B	+7.10	Shikimate 5-dehydrogenase	-4.83

GBP	+6.43	similar to GHITM protein	-4.28

C-C chemokine receptor 11 like	+6.42	similar to keratin	-3.39

GAL7	+6.04	TAC1	-3.38

LITAF	+5.82	similar to ALS2	-3.17

Cytochrome b	+5.30	TMEM22	-2.95

C3AR1	+5.09	HVCN1	-2.90

similar to regulator of G-protein signaling 1	+5.00	MMP13	-2.83

MX	+4.96	similar to EPHX1 protein	-2.66

LYZ	+4.40	VSX1	-2.55

GPR174	+4.21	GOLGB1	-2.54

IL21R	+4.11	FMO6	-2.47

similar to MSTP055	+3.99	HIST2H3C	-2.46

ISG12-2	+3.91	similar to BTBD7	-2.45

^a: ^DE genes without gene information, from cDNA clones and of hypothetic proteins were deleted. The whole list of DE genes for all SL samples before deletion is available upon request

^b: ^NV indicates that feather samples were from SL chickens that never developed vitiligo; EV, AV, and CV indicates growing feathers were from SL chickens with vitiligo (SLV) before, during and after complete loss of pigmentation

^c: ^numbers in the body of the table are fold changes relative to gene expression levels in samples from BL (the parental line) chickens. For comparison purpose, the data obtained from the microarray analysis for down-regulated genes (< 1) were expressed as fold changes by calculation: the ratio of 1 to the number (e.g. for DE value of 0.12 the fold change would be 8.3; i.e. 1/0.12). Up- and down-regulated DE genes were indicated by a positive and negative sign, respectively

The magnitude of the fold change of the top 10% up-regulated genes was lowest in NV samples, intermediate in EV and CV and highest in AV samples. The three up-regulated DE genes with the highest expression were immunoglobulin J chain (*IGJ*), acidic chitinase (*CHIA*) and T cell receptor (*TCR*) delta in NV; chemokine ligand 13 (*CXCL13*), guanylate binding protein (*GBP*) and lipopolysaccharide-induced TNF factor (*LITAF*) in EV; sodium channel alpha subunit, *IGJ *and *CXCL13 *in AV; and, *IGJ*, POU class 2 associating factor 1 (*POU2AF1*) and myeloid antimicrobial peptide 27 in CV. The magnitude of the fold change of down-regulated DE genes was similar in NV, EV, and AV samples, but higher in CV samples due to marked decreased expression of solute carrier family 24 member 5 (*SLC24A5*), tyrosinase (*TYR*), and matrix metalloproteinase 115 (*MMP115*).

Some of the top 10% DE genes were shared by more than two SL samples, although their expression-levels varied. Up-regulated DE genes shared by EV, AV and CV samples (SLV samples) included *LITAF*, tumor necrosis factor superfamily (*TNFSF*) 13B, interleukin 21 receptor (*IL21R*), complement component 3a receptor 1 (*C3AR1*) and sodium channel alpha subunit, with higher expression levels observed in AV than in EV and CV samples. Down-regulation was noticed in lipoprotein *VSAF*, matrix metallopeptidase 9 (*MMP9*), *MMP13*, growth hormone induced transmembrane protein (*GHITM*), transmembrane protein 22 (*TMEM22*), keratin, flavin-containing monooxygenase 6 (*FMO6*), and histone cluster 2H3C (*HIST2H3C*) in all SL samples (NV and SLV samples), with lipoprotein *VSAF *exhibiting the largest decrease in expression. Other down-regulated DE genes observed in SLV samples were parathyroid hormone (*PTH*) and tachykinin precursor 1 (*TAC1*), with larger decreases in *PTH *expression in AV and CV samples than in EV samples. Expression of *SLC24A5, MMP115*, and *TYR*, and genes for crystalline alphaB (CRYAB), Shikimate 5-dehydrogenase and amyotrophic lateral sclerosis 2 (ALS2) were commonly depressed in AV and CV samples relative to BL controls.

### Within SL comparisons of DE genes in NV, EV, AV and CV samples

JMP genomics 4 analysis revealed 206 DE genes for within SL comparisons, which resulted in 88 DE genes after deletion of unknown genes (without information, from cDNA libraries and clones and related to hypothetical proteins). The 88 DE genes could be roughly divided into 4 functional groups which were immunity-, melanocyte-, stress-related and others (Table 2) based on functions of their corresponding proteins. More than half of the DE genes were immune system related, including molecules of both innate and adaptive branches of the immune system with more DE genes relating to innate than adaptive immune response activities (Table 2). The group of DE genes related to stress contained DE genes associated with cellular stress and apoptosis.

**Table 2:** Comparison of DE genes between SL samples (within SL comparison)*

GeneName/Symbol	Accession #	NV	EV	AV	CV
**Immunity-related**					

**Innate immunity-related**					

similar to MD-2	BX932484	1.56^d^	5.47^b^	8.02^a^	3.47^c^

LYZ	CR390743	1.40^c^	4.70^b^	8.64^a^	4.22^b^

lymphotactin	BX930561	0.93^b^	2.29^a^	2.66^a^	2.18^a^

Myeloid antimicrobial peptide 27	DQ092352	0.63^b^	0.78^b^	8.71^a^	12.67^a^

Estrogen responsive finger protein	BX950599	0.95^c^	2.80^a^	2.94^a^	1.91^b^

GAL7	AY621309	1.00^b^	0.89^b^	6.20^a^	5.59^a^

GBP	X92112	2.56^c^	11.57^a^	9.87^a^	6.09^b^

Very large inducible GTPase-1	TC195275	0.44^c^	2.70^a^	1.99^a^	0.80^b^

IRF8	L39767	1.09^c^	1.66^b^	2.79^a^	2.43^a^

IRF1	L39766	1.58^c^	5.72^a^	5.22^a^	2.52^b^

TLR15	DQ267901	1.23^c^	5.49^a^	7.62^a^	2.59^b^

C3	U16848	0.99^c^	1.57^b^	2.23^a^	0.99^b^

C-C chemokine receptor 11 like	TC224438	1.51^c^	4.49^b^	6.96^a^	6.00^ab^

Chemokine ah294	TC190676	1.51^c^	6.08^a^	6.84^a^	2.78^b^

Cytokine	L34552	1.19^c^	3.74^ab^	4.16^a^	2.61^b^

CXCR4	AF294794	1.45^c^	1.99^b^	3.86^a^	2.06^b^

IL15	AF152927	0.91^c^	1.31^b^	1.96^a^	1.26^b^

IL18	AY775780	1.14^d^	2.22^b^	3.74^a^	1.68^c^

Beta-defensin	CO769187	0.96^c^	1.12^c^	10.25^b^	17.27^a^

TNFSF13B	AJ721035	2.05^c^	8.89^b^	14.91^a^	6.63^b^

chemokine (C-C motif) receptor 8	AJ720982	1.38^c^	3.40^a^	4.07^a^	2.39^b^

phosphoinositide-3-kinase, regulatory subunit 5	AJ720866	1.19^c^	1.76^b^	2.68^a^	1.57^b^

CD200 receptor 1	BX935064	1.65^a^	0.71^c^	1.27^ab^	0.96^b^

C3AR1	AJ720748	2.02^c^	6.38^ab^	9.49^a^	4.73^b^

MALT1	AJ851524	1.01^c^	2.56^b^	3.81^a^	2.45^b^

CD39	CR385269	0.89^c^	1.59^b^	2.11^a^	1.45^b^

FKBP1B	BU107658	0.73^a^	0.39^b^	0.21^c^	0.27^c^

similar to IMMUNE RESPONSIVE PROTEIN 1	AJ720739	1.32^c^	6.04^a^	10.21^a^	3.29^b^

hematopoietic cell-specific Lyn substrate 1	AJ719284	0.86^c^	2.24^ab^	3.36^a^	1.95^b^

CD83	CR733120	1.71^d^	3.71^b^	5.59^a^	2.73^c^

IRF4	AF320331	1.22^b^	1.38^b^	3.07^a^	3.60^a^

similar to Cathepsin L	BX934408	1.25^c^	2.34^b^	3.43^a^	1.80^b^

**Adaptive immunity-related**					

CR2	AJ720954	1.16^c^	5.04^b^	7.79^a^	4.47^b^

POU2AF1	AJ720333	1.95^c^	4.00^b^	14.79^a^	16.84^a^

BTK	AJ719782	0.93^c^	2.18^b^	3.25^a^	2.16^b^

Mucolipin 2	TC215961	1.36^d^	5.33^b^	9.59^a^	3.25^c^

PIK3AP1	AF315784	1.29^c^	2.57^b^	4.57^a^	2.92^b^

CD5	Y12011	0.86^c^	2.06^ab^	2.61^a^	1.69^b^

TRAF3-interacting JNK-activating modulator	TC210615	1.64^c^	3.07^b^	4.98^a^	2.45^b^

SPI1	Y12225	1.24^c^	4.08^a^	5.23^a^	2.22^b^

LAT2	BX950412	0.88^c^	1.25^b^	2.89^a^	2.15^a^

IL2RG	AJ419896	1.56^c^	2.54^b^	4.22^a^	2.46^b^

LCP1	AJ719624	1.05^c^	1.89^b^	2.54^a^	1.54^b^

CD8 beta chain	Z26484	1.31^c^	3.52^a^	3.24^a^	2.11^b^

TCR beta chain	M81150	1.21^c^	2.55^b^	3.71^a^	2.71^ab^

MHCII	U76305	1.21^c^	2.37^b^	3.77^a^	1.84^b^

B2M	AB178593	0.98^c^	1.77^b^	2.94^a^	1.66^b^

TAP2	AJ843262	1.19^c^	4.40^a^	4.26^a^	2.28^b^

GZMA	AJ544060	2.48^b^	6.86^a^	6.79^a^	2.96^b^

**Melanocyte-related**					

TYR	AB023291	1.22^a^	1.00^a^	0.40^b^	0.07^c^

TRP1	AF003631	1.03^a^	1.03^a^	0.24^b^	0.03^c^

MMP115	D88348	0.92^a^	0.46^b^	0.17^c^	0.07^d^

SLC24A5	TC191950	0.93^a^	0.50^b^	0.13^c^	0.05^d^

SLC24A2	AF177985	1.08^a^	0.78^b^	0.45^c^	0.48^c^

GPR143	BX950829	1.16^a^	0.74^b^	0.26^c^	0.11^d^

V-ATPase C2 subunit	TC218700	0.83^a^	0.62^ab^	0.50^b^	0.12^c^

Shikimate 5-dehydrogenase	TC216663	0.86^a^	0.20^d^	0.29^c^	0.51^b^

**Stress-related**					

NPY	M87294	1.03^a^	0.13^d^	0.41^c^	0.94^b^

CRYAB	U26661	1.14^a^	0.18^c^	0.24^b^	0.56^b^

BLVRA	BU468223	0.96^c^	1.27^c^	1.80^b^	2.91^a^

GSTA1	L15386	1.19^c^	0.73^c^	2.00^a^	1.40^b^

Gp91-phox	TC214833	1.50^d^	3.01^c^	10.02^a^	5.43^b^

CD163	CR406525	1.39^c^	2.18^b^	3.93^a^	1.80^c^

neutrophil cytosolic factor 1	AJ719555	0.90^d^	2.57^b^	4.64^a^	1.49^c^

Cytochrome b	TC220424	1.38^b^	5.06^a^	6.91^a^	5.14^a^

Acetyl-CoA carboxylase 2	TC206581	1.08^d^	3.58^a^	2.28^b^	1.59^c^

LITAF	AB058634	1.41^d^	5.48^c^	14.05^a^	9.29^b^

CARD11	AJ851540	1.03^c^	2.63^b^	4.62^a^	2.01^b^

Gasdermin 1	AJ721093	0.83^c^	2.14^a^	2.89^a^	1.27^b^

TRAF5	AJ720372	1.30^c^	2.65^b^	4.25^a^	2.35^b^

PDCD1	CR390246	1.44^d^	3.91^a^	5.67^a^	2.38^c^

**Others**					

GJA5	M35043	1.01^a^	0.73^b^	0.51^c^	0.43^c^

TMEM9	TC216868	0.91^a^	0.70^b^	0.51^c^	0.32^d^

ATP synthase F0 subunit 8	TC213211	1.20^c^	3.40^a^	3.30^a^	2.13^b^

Novel Ras family member protein	TC196126	1.28^c^	2.64^ab^	3.31^a^	2.23^b^

KCNMB1	AF077369	0.83^c^	1.78^b^	2.80^a^	2.51^a^

BTN1A1	AY847576	0.81^c^	1.79^b^	2.53^a^	1.84^b^

TF	AB215094	0.97^d^	2.41^b^	3.83^a^	1.54^c^

GPR174	AJ719761	2.04^c^	4.02^b^	6.58^a^	3.92^b^

LIMD2	AJ721104	0.93^c^	1.62^b^	2.31^a^	1.54^b^

TSPAN15	AJ720604	1.00^c^	2.01^b^	3.27^a^	1.69^b^

LOC423781	CR405911	0.86^c^	2.42^b^	4.26^a^	2.37^b^

ASAHL	BX950518	1.47^c^	3.60^b^	6.29^a^	2.87^b^

SYT12	BX936066	0.80^a^	0.59^b^	0.33^c^	0.42^c^

AKAP12	BX932296	0.96^a^	0.71^b^	0.46^c^	0.39^c^

AGR2	BX934938	0.88^a^	0.67^b^	0.41^c^	0.38^c^

similar to Rho-GTPase-activating protein 6	CR407051	1.08^d^	2.50^b^	3.66^a^	1.44^c^

FBP2	BU312699	1.06^a^	0.63^b^	0.46^c^	0.37^c^

*: NV indicates that growing feather samples were from SL chickens that never developed vitiligo; EV, AV, and CV indicate that growing feather samples were collected from SL chickens with vitiligo (SLV) within 1 week before SLV onset, during active depigmentation and at least one week after complete loss of pigmentation, respectively. Normalized microarray data were filtered to include only fluorescence values above 100 and analyzed by JMP genomics 4 to determine differences in NV, EV, AV and CV gene expression levels. Values in the body of the table were mean expression (n = 3) relative to BL samples, where numbers > 1 and < 1 represents up-regulated and down-regulated expression, respectively. Means without a common letter were significantly different at P ≤ 0.05

Compared to SLV samples, the majority of DE genes in NV samples had the lowest expression level except for all DE genes related to melanocyte functions (*MMP115, TRP1, TYR *and *SLC24A5, SLC24A2, GRP143*, V-ATPase C2 subunit and Shikimate 5-dehydrogenase). Additionally, DE genes related to stress (*NPY *and *CRYAB*) and intra/inter-cellular transport [gap junction protein alpha 5 (*GJA5*), transmembrane protein 9 (*TMEM9*) and synaptotagmin 12 (*SYT12*)], exhibited the highest expression in NV samples (Table 2). For most DE genes, the expression levels in EV and CV samples were intermediate to their expression in NV and AV samples. All genes related to immune system activities had the highest expression levels in AV compared to NV, EV and CV samples (Table 2). The expression levels of DE genes in CV samples tended to be comparable to EV and/or NV samples. Expression of melanocyte-related DE genes (i.e. *MMP115, TYR, TRP1, SLC24A2, SLC24A5*, V-ATPase C2 subunit), however, was decreased significantly in AV and reached the lowest expression in CV samples compared to those from NV and EV samples. DE genes in the others category, e.g. intra/inter-cellular transport (*GJA5 *and *TMEM9)*, also exhibited the lowest expression levels in CV compared to NV, EV and AV samples (Table 2).

### Ingenuity Pathway Analysis (IPA) for SL samples

The three major output components provided by Ingenuity Pathway Analysis (IPA) are function, network and pathway interpretations (IPA, Version 9.0; Ingenuity Systems^®^; http://www.ingenuity.com). The functional analysis identifies biological functions and/or diseases that are most closely related to the data set. Network interpretations are algorithmically generated by overlying genes from a data set onto a global molecular network developed from information contained in Ingenuity's Knowledge Base. Canonical pathway analysis identifies the pathways from the Ingenuity Pathways Analysis library of canonical pathways that most significantly fit the data set (IPA, Version 9.0; Ingenuity Systems^®^; http://www.ingenuity.com)

### Functional analysis

**Figure 1:**
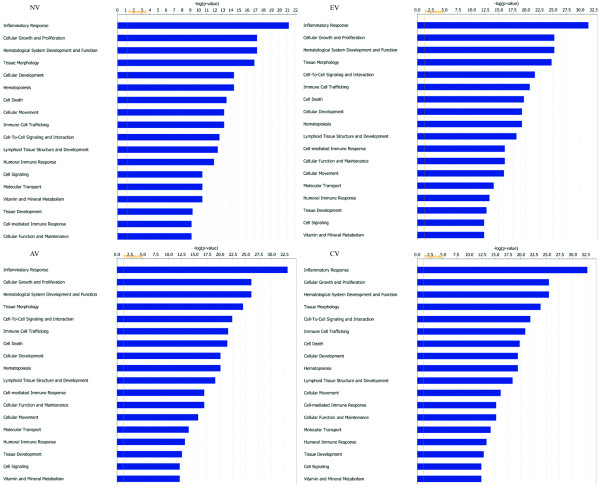
**Graphical demonstration of associated functions from Ingenuity Pathway Analysis (IPA) of differentially expressed (P ≤ 0.05; DE) genes for NV, EV, AV, CV relative to BL samples**. The functional analysis identifies biological functions and/or diseases that are most significant to the data set. Molecules from the DE gene dataset that are associated with biological functions and/or diseases in Ingenuity's Knowledge Base were considered for the analysis. Right-tailed Fisher's exact test was used to calculate a P-value determining the probability that each biological function and/or disease assigned to that data set is due to chance alone. The y-axis displays the functional categories that are identified in analyses. The x-axis demonstrates the significance which is the value of -log (P). Functions are listed from most significant to least and the orange vertical line denotes the cutoff for significance (P-value of 0.05). For each analysis, only the top 18 functional categories are displayed due to large size of the data files.

IPA analysis provided similar composition and ranking of functions for all SL samples (Figure 1). Common functions not only included those related to normal biological functions, such as cellular-function and -development and lipid metabolism, but also those associated with a variety of diseases, e.g. immunological disease, neurological disease, dermatological disease/condition and cancer. Among them, the inflammatory response was the most significant (lowest *P *values which were due to chance alone) functional interpretation and the first function identified for all SL groups. However, for any particular common function identified in the IPA analysis of SL samples, the associations were made with higher confidence (lower *P *values) for DE genes in SLV (EV, AV and CV) samples than in NV samples. Unique functional interpretations of NV DE genes included digestive system development and function as well as organismal survival. Based on function analysis of DE genes identified by within SL comparisons, amino acid/carbohydrate metabolism, energy production, protein folding and developmental disorder were uniquely demonstrated, however with very low confidence.

### Network generation

**Figure 2:**
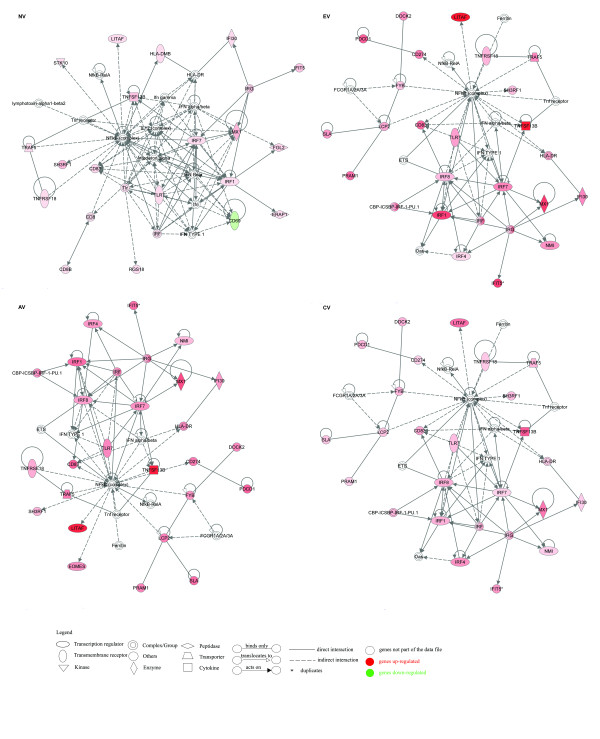
**Network #1 obtained by Ingenuity Pathway Analysis (IPA) of differentially expressed (P ≤ 0.05; DE) genes for NV, EV, AV, CV relative to BL samples**. DE genes from the data sets were overlaid onto a global molecular network developed from information contained in Ingenuity's Knowledge Base to algorithmically generate networks that graphically represent relationships between molecules. Molecules are represented as nodes, and the biological relationship between two nodes is represented as an edge (line). The intensity of the node color indicates the degree of up- (red) or down- (green) regulation. Nodes are displayed using various shapes that represent the functional class of the gene product. Edges are displayed with various labels that describe the nature of the relationship between the nodes.

Network output provided number wise more networks for DE genes in SLV samples than for DE genes in NV samples or DE genes identified by within SL comparison. Functional analysis of networks demonstrated that functions for the NV samples and the within SL comparison group were part of the functional networks identified for DE genes in SLV samples. In addition to shared functions between all groups, humoral immune response and immunological disease were only present in EV samples, and cell-mediated immune response was unique to CV samples. The 1^st ^network for NV samples was associated with functions of cellular movement, immune cell trafficking and cell morphology; for EV samples, functions of inflammatory response, antimicrobial response and humoral immune response; and for AV and CV samples, inflammatory response, cellular growth and proliferation and hematological system development and function, and cellular development. These number one networks are illustrated in Figure 2.

### Canonical pathway analysis

Pathway composition was similar for DE genes in SL samples, with higher levels of similarity between DE genes for SLV samples. For a specific common pathway higher significance [suggested by a higher ratio (number of molecules from the data set divided by the total number of molecules in the pathway obtained from the Ingenuity's Knowledge Base) and a lower *P *value] was exhibited for SLV samples than for NV samples. Pathway analysis of the DE data-set generated by within SL comparison had numerically the most pathways which included all pathway components for all SL samples. The pathway of communication between innate and adaptive immune cells was most significant, constituting the number-one pathway for all SL samples and within SL comparison.

### Microarray validation by qRT-PCR

Microarray data were validated by qRT-PCR based on comparable gene expression levels and similar expression trends of selected targets throughout SLV development (Table 3). Fold changes in complement component receptor 2 (*CR2*) and *POU2AF1 *from qRT-PCR, however, were noticeable higher than those from microarray.

**Table 3:** Relative expression of DE genes in SL samples^a ^from microarray vs. qRT-PCR analysis^b^

		NV		EV		AV		CV	
**GeneSymbol**	**Accession #**	**Micro-array**	**RT-PCR**	**Micro-array**	**RT-PCR**	**Micro-array**	**RT-PCR**	**Micro-array**	**RT-PCR**

IRF1	L39766	1.58	1.73	5.72	7.32	5.22	8.08	2.52	3.46

TLR15	DQ267901	1.23	1.47	5.49	12.54	7.62	18.41	2.59	3.66

CR2	AJ720954	0.21	9.88	2.33	54.92	2.96	174.26	2.16	62.87

POU2AF1	AJ720333	0.96	3.63	2	18.32	3.89	61.51	4.07	67.04

B2M	AB178593	0.98	1.28	1.77	3.46	2.94	4.82	1.66	3.01

CRYAB	U26661	1.14	1.41	0.18	0.53	0.24	0.19	0.56	0.09

NPY	M87294	1.03	0.79	0.13	0.49	0.41	0.1	0.94	0.004

TRAF5	AJ720372	0.37	1.44	1.4	4.61	2.09	6.39	1.24	3.99

LITAF	AB058634	1.41	1.42	5.48	13.21	14.05	21.88	9.29	11.23

TYR	AB023291	1.22	1.12	1	0.8	0.4	0.26	0.07	0.001

TRP1	AF003631	1.03	1.25	1.03	0.51	0.24	0.04	0.03	0

^a, ^SL samples included NV, EV, AV and CV samples which were from SL chickens that never developed vitiligo; from vitiliginous SL chickens within 1 week before SLV onset, during active depigmentation and at least one week after complete loss of pigmentation, respectively

^b, ^Gene expression in NV, EV, AV and CV was presented by fold changes relative to expression in BL samples in microarray and in qRT-PCR, where fold-change was determined by the delta delta Ct method. The same RNA pools for NV, EV, AV, CV and BL samples used in the microarray study were reverse transcribed to cDNA, which was subjected to qPCR with GAPDH as the endogenous control gene and cDNA from BL sample as the calibrator. All values are fold change means of three replications analyzed by JMP Genomics 4 for microarray and SYSTAT for qRT-PCR data

## Discussion

The Smyth line (SL) of chicken is an excellent avian model for human autoimmune vitiligo [4-6], which is a multifactorial disease of complex etiology resulting in loss of melanocytes in the skin. In the current study, we took advantage of the unique attributes of the SL chicken model (outlined in the Introduction) and the power of gene expression analysis by microarray to investigate the pathomechanism of vitiligo in SL chickens (SLV). To our best knowledge, this is the first paper using microarray analysis to study transcriptomic expression profiles in evolving vitiligo lesions. Expression levels of genes from this microarray study were validated based on their comparability to those from qRT-PCR (Table 3), which was suggested to have higher detection sensitivity than microarray [26]. While higher expression was observed for *CR2 *and *POU2AF1 *by qRT-PCR than with microarray, their expression trends were the same with both methodologies, supporting the validity of the microarray study. The expression difference may be due to the upper fluorescent detection limit of the microarray scanner. Differentially expressed (DE) genes identified by microarray analysis and the interpretations by Ingenuity Pathway Analysis http://www.ingenuity.com support the multifactorial nature of vitiligo. For the sake of simplicity, the discussion will focus on observations regarding the involvement of the immune system, melanocyte biology, redox status, neurology, apoptosis and other physiopathological factors identified to play a role in the spontaneous development of SLV.

### Immune system

The autoinflammatory/autoimmune nature of SLV was supported by expression profiling (Table 1 and 2) and IPA functional interpretations of DE genes (e.g. inflammatory response and immunological disease) (Figure 1 and 2). DE genes of both T and B cell markers (Table 1 and 2) and functions of humoral and cell-mediated immune response (Figure 1 and 2) in the current study are in line with previous studies demonstrating involvement of both types of immune responses in melanocyte loss in the etiology of SLV [6,10,12,16]. Furthermore, it appears that the humoral immune response was particularly important in early SLV development, since DE genes in EV samples were significantly (1^st ^network) associated with this function (Figure 2). In addition, the humoral immune response also appeared important in SLV progression (AV) and complete depigmentation (CV) as supported by significantly up-regulated DE genes related to B cell development and/or activation such as *IGJ *(immunoglobulin J chain), *POU2AF1 *(a protein essential for the B cell response and germinal center formation), *BTK *(Bruton's tyrosine kinase), mucolipin 2 (a target for BTK in B cells), *SPI1 *(a lymphoid specific enhancer involved in B cell differentiation and activation), and *PIK3AP1 *(B cell activation) in these samples (Table 1 and 2). On the other hand, DE genes associated with the cell-mediated immune response were identified only in CV samples and with lower significance than DE genes associated with the humoral immune response in EV samples. A more relevant role of the humoral immune response in the pathomechanism of SLV is contradictory to the demonstrated more direct role of the cell-mediated immune response in this disorder [6,16]. Confirmation of this finding will definitely impact future direction of vitiligo research.

Aside from involvement of adaptive immunity in SLV, it is apparent that innate immunity also plays an important role in SLV development since the majority of immune-related DE genes were derived from this branch of the immune system (Table 1 and 2). Innate immunity has not received much attention in vitiligo studies in either the SL animal model or human patients. Elevated expression of genes for chemokines (e.g. *CXCL13, CCL19*), pattern recognition receptors (e.g. *MMR, TLR15, MD-2 *and *PRAM-1*), TNF and type 1 and 2 interferon cytokines (e.g. *LITAF, TNFSF13B, ISG12-2, ITIF5, IRF1 *and *IRF8*) (Table 1 and 2) suggested both anti-bacterial and anti-viral activity especially in early and active stages of SLV (EV and AV). The complement system also appears to participate in SLV development and progression as supported by significantly increased expression of genes for C3, C3AR1 and CR2 in AV samples (Table 1 and 2). Functional analysis of networks by IPA revealed that DE genes identified in EV and AV samples were significantly associated (1^st ^and 3^rd ^network) with the functions of anti-microbial response and infectious disease, respectively. These observations suggest a potential role of the innate immune response as a precipitating and effector factor in the development of the melanocyte-specific adaptive immune response in SLV. Initiation of antimicrobial activity in SLV is in agreement with the strong association of live herpesvirus of turkey administration at hatch (routine vaccination to protect SL and BL chickens from Marek's disease virus) as a reliable environmental trigger of SLV expression in vitiligo-susceptible SL chickens [19]. Additionally, as innate immunity is also initiated in response to cellular stress, the observed alterations in melanocyte biology and redox status (see below) may also be responsible for activation of innate immune responses in SLV [27].

### Melanocyte biology

It was previously shown that intrinsic abnormalities of melanocytes, the target cell in vitiligo, were present in SL chickens compared to BL and LBL (vitiligo-resistant, similar plumage color pattern control) controls [7,28]. In the current study, melanocyte loss appeared to be preceded by disturbances in melanosome structure, intracellular molecule transport and tyrosine synthesis as demonstrated by decreased expression of *MMP115, SLC24A2, SLC24A5, GPR143*, and Shikimate 5-dehydrogenase in EV samples (Table 1). Dysfunction and loss of melanogenesis became obvious in AV and CV samples, as suggested by significantly decreased gene expression in *TYR *and *TRP1 *in these samples compared to NV and EV samples (Table 2). This finding is consistent with findings in human studies, where down-regulation of TYR and TRP1 was revealed in lesional skin compared to non-lesional skin of vitiligo patients and skin from healthy donors [29-31].

### Redox status

Oxidative stress was previously demonstrated in vitiligo patients [32-35] and in SLV chickens [18]. In humans, oxidative stress involvement was further supported by the repigmenting effect of antioxidant substances in the treatment of vitiligo lesions [36-38]. In line with findings from these studies, there were DE genes for all SL samples and those generated from within SL comparison identified by IPA to be associated with the function of free radical scavenging (including functional annotation of synthesis/production of oxidative species). NV samples had similar DE gene expression for both pro- and anti-oxidative proteins relative to the BL control samples whose expression was considered as the value of 1 (Table 2). Up-regulation of these DE genes in SLV samples, especially AV samples (Table 2) suggested association of SLV development with disturbances in redox status. Among these, remarkably elevated expression was noticed for Gp91-phox and cytochrome b (Table 2), suggesting an important role of these proteins in changing the redox status in SLV chickens. Gp91-phox is a subunit of NADPH oxidase containing the heme binding site important in reactive oxygen species (ROS) production [39]. The source of NADPH-dependent ROS production in vitiligo development is not clear, however, after activation, phagocytes and B cells are capable of NAPDH-dependent release of ROS [40,41]. With more DE genes related to B cell than phagocyte development and/or activation in the current study (Table 1 and 2) and the greater B cell than macrophage infiltration in feather tissues at all vitiligo states reported by Shi and Erf [6], it is likely that B cells could be an important source of ROS in SLV development. As revealed by IPA, decreased transmembrane potential of mitochondria and the mitochondrial membrane was one of the many functional interpretations of DE genes in EV, AV and CV samples suggesting mitochondrial involvement in ROS production. Elevated expression of cytochrome b and acetyl-CoA carboxylase 2, which is important in fatty acid oxidation, in EV, AV and CV compared to NV samples points to mitochondrial production of ROS probably due to premature electron leakage of oxygen at the location of cytochrome b in the electron transport chain [42].

### Neurology

Neural involvement in vitiligo was supported by appearance of localized vitiligo lesions following nerve damage in affected skin and by morphological changes in terminal cutaneous nerves in vitiligo patients [43]. In line with observations in humans, neurological disease was presented as one of the functional interpretations by IPA for DE genes in SL samples and DE genes identified based on within SL comparisons in the current study. In addition, expression of *TAC1 *(gene for neurotransmitter substance P, neurokinin A, neuropeptide K and neuropeptide gamma), *KCNE1L *(a protein capable of regulating neurotransmitter release and neuronal excitability), *ALS2 *(plays a role in axon and dendrite development) and *NPY *(neuropeptide Y) were decreased in EV, AV, and CV samples compared to NV samples (Table 1 and 2), supporting neural involvement in SLV development. NPY expression was also investigated in human studies; the results, however, were not consistent [44,45] and differed from the decreased expression with SLV progression in the current study. Other studies revealed an increased number of nerve growth factor receptor-, calcitonin gene-related peptide- and protein gene product 9.5-positive nerve fibers in involved skin from vitiligo patients compared to healthy controls [45,46]. Moreover, increased serum and urinary levels of catecholamines and their relative metabolites were found in vitiligo patients, especially in early and active stages of vitiligo, than in controls [47-49]. It was speculated that a role of catecholamines in vitiligo onset was due to their potential to undergo oxidation, resulting in formation of quinones, semiquinone radicals and oxyradicals [47,49].

### Apoptosis

While the mechanism by which melanocytes are destroyed in vitiligo is still in debate, it has been suggested that they disappear via apoptosis (presumably initiated by cytotoxic T cells) rather than necrosis [50]. In the current study, significant up-regulation of apoptosis-related DE genes, especially in AV compared to NV, EV and/or CV samples (Table 2), indicated a close relationship of apoptosis with SLV development. These apoptosis-related up-regulated DE genes included *GZMA, LITAF *(induced by p53 and has been implied in p53 induced apoptosis), caspase-associated recruitment domain, member 11 (*CARD11*, a positive regulator of cell apoptosis), gasdermin 1 (involved in apoptosis induction), TNF receptor associated factor 5 (*TRAF5*) and programmed cell death 1 (*PDCD1*) (Table 2). In addition, participation of apoptosis in SLV etiology was also supported by the IPA association of functions such as cell death (Figure 1) and pathways of apoptosis signaling and cytotoxic T cell-mediated apoptosis of target cells with DE genes in SL samples and DE genes identified by within SL comparison. These findings are consistent with elevated levels of apoptotic (TUNEL+) cells in the melanocyte region of growing feathers from vitiliginous SL chickens (particularly during active depigmentation) compared to non-vitiliginous SL, BL and LBL controls [17]. The mechanism of apoptosis was also indicated by decreased levels of anti-apoptotic protein Bcl2 in lesional skin compared to nonlesional skin of vitiligo patients [51,52].

### Others

The decreased expression of the gene for keratin especially in EV and AV compared to NV samples (Table 1) may suggest disturbance in keratinocyte function in early stages of SLV development. Since keratinocytes play important roles in supporting melanocyte growth and regulating melanogenesis in melanocytes [53], keratinocyte malfunction can have detrimental effects on melanocyte functions. Constant and concurrent down-regulation in matrix metalloproteinase *MMP9 *and *MMP13 *in SL chickens could be due to aberrant keratinocyte function, based on the observations in human studies that keratinocyte-derived MMP9 was important in repigmentation by enhancing melanocyte migration [54,55]. Three DE genes *TMEM9, TMEM22 *and sorting Nexin 10 may suggest abnormal inter/intracellular molecule/organelle trafficking in SLV chickens. The reason for noticeably decreased expression in lipoprotein VSAF in SL chickens compared to BL is not clear due to lack of functional information on this protein. Significant down-regulation of parathyroid hormone (*PTH*) in EV, AV and CV compared to NV samples may suggest a possible role of *PTH *in SLV expression as its biological function is to increase serum calcium levels and vitamin D synthesis, both of which are important in melanogenesis.

## Conclusions

The multifactorial etiology of autoimmune SLV is supported by the DE gene profiles from the microarray study and by their interpretations by IPA. Basically, in susceptible SL chickens SLV may occur primarily through melanocyte-specific immune destruction of aberrant melanocytes under certain pathobiological circumstances where pre-existing "danger" signals and oxidative stress may act as essential triggers.

## Competing interests

The authors declare that they have no competing interests.

## Authors' contributions

FS performed the sample collection, carried out the experiment, collected the microarray data, participated in statistical analysis and drafted the manuscript. BWK participated in the design of the study and performed the statistical analysis. JJS led the statistical analysis. JYL participated in the microarray experiment, data collection and statistical analysis. RLD helped with the sample collection and coordination of animal husbandry and laboratory procedures. GFE conceived of the study, participated in its design and coordination and manuscript preparation. All authors read and approved the final manuscript.
